# Supplementary material for: MAP3Kinase-dependent SnRK2-kinase activation is required for abscisic acid signal transduction and rapid osmotic stress response
Source: Nat Commun. 2020 Jan 2;11:12. doi: 10.1038/s41467-019-13875-y (PMC6940395; doi:10.1038/s41467-019-13875-y)
Supplement: Supplementary file 1 — Supplementary Information [file 41467_2019_13875_MOESM1_ESM.pdf]

Supplemental Information for

MAP3Kinase-dependent SnRK2-kinase activation is required for abscisic acid signal transduction and rapid osmotic stress response

Takahashi et al.

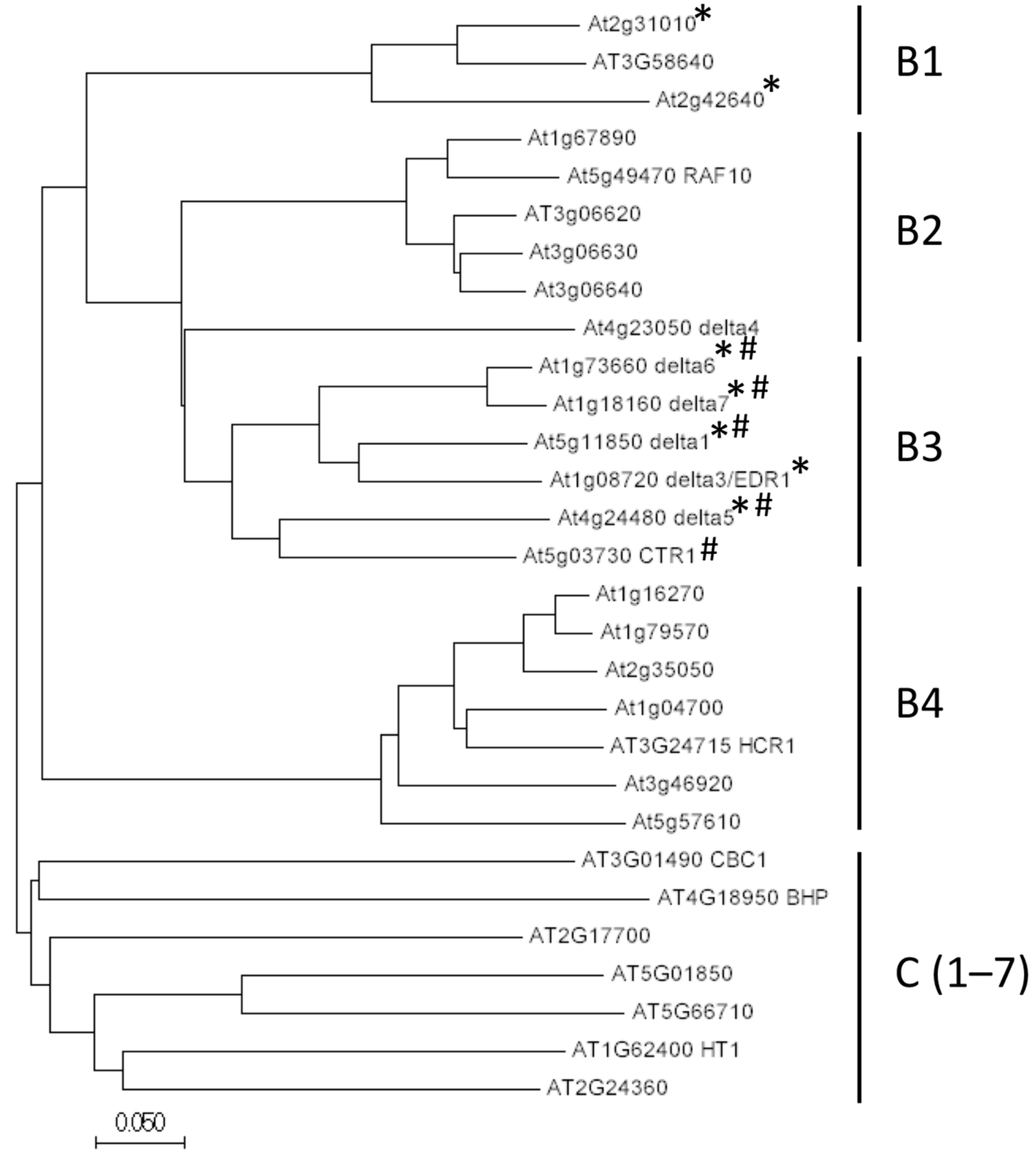

Supplementary Figure 1 | Phylogenetic tree of *Arabidopsis* Raf-like MAPKK kinases.

All M3Ks in subgroup B and selected M3Ks in subgroup C1-7 are shown[1]. # and * indicates genes targeted by the *amiR-ax1117*, and the *m3k* amiRNA, respectively.

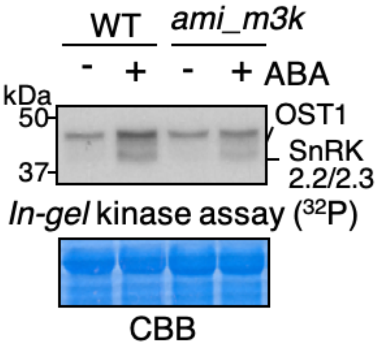

Supplementary Figure 2 | Biological replicate example of SnRK2 activities in *m3k* amiRNA line.
Replicate of *in-gel* kinase assay using *m3k amiRNA* line and WT (Col-0 accession) is shown. See Figure 1d for an independent experiment.

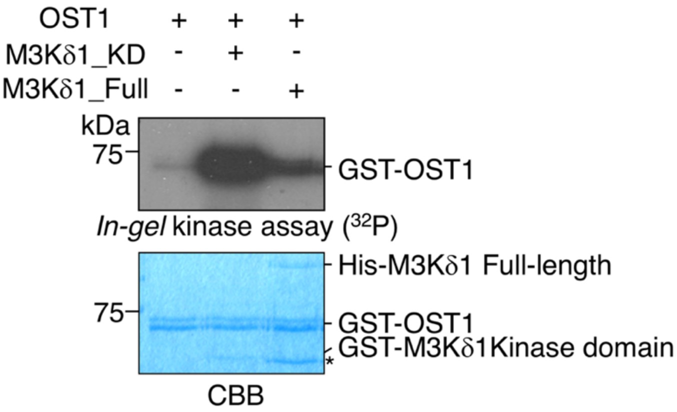

Supplementary Figure 3 | Full-length M3Kδ1 activates OST1/SnRK2.6 *in vitro*.

Recombinant GST-OST1 and full-length His-M3Kδ1 (M3Kδ1_Full) or GST-M3Kδ1_KD (kinase domain) were incubated in the presence of ATP for 30 min. OST1/SnRK2.6 activity was measured by *in-gel* kinase assays. Note that the truncated kinase domain (M3Kδ1_KD) has a higher activity than the full length M3Kδ1 protein (M3Kδ1_Full). CBB shows loading control. The band labeled by an asterisk showing a similar mobility to the GST-M3Kδ1_KD in the right lane may be a degradation product of His-M3Kδ1.

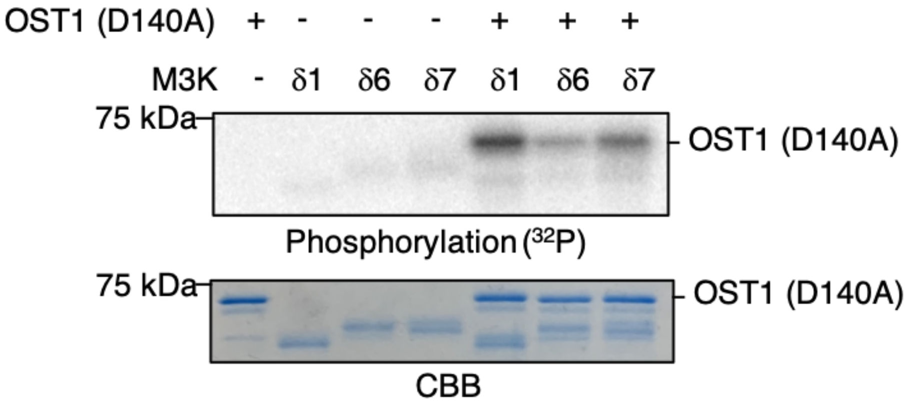

Supplementary Figure 4 | M3Kδ1, δ6 and δ7 directly phosphorylate OST1/SnRK2.6. Recombinant kinase inactive GST-OST1/SnRK2.6 (D140A) protein was incubated with M3Kδ1, M3Kδ6 or M3Kδ7 kinase domains, and *in vitro* phosphorylation assays were performed with ^{32}P-ATP.

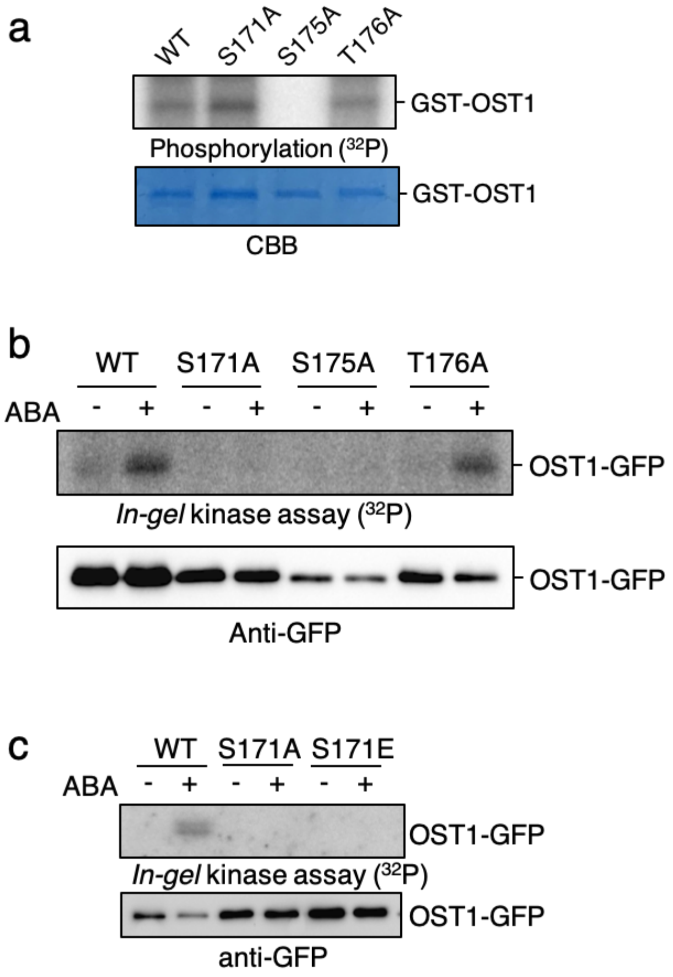

Supplementary Figure 5 | Ser-171 is important for ABA-activation of OST1/SnRK2.6 but not for *in vitro* protein kinase enzyme activity.

a, Recombinant GST-OST1/SnRK2.6 proteins carrying S171A, S175A or T176A mutation were used for *in vitro* autophosphorylation. OST1/SnRK2.6 (S175A) has no kinase activity[2]. **b**, OST1/SnRK2.6-GFP variants (WT, S171A, S175A or T176A) were transiently expressed in *Arabidopsis* mesophyll cell protoplasts. Protoplasts were incubated in 10 μM ABA for 15 min. OST1/SnRK2.6 protein kinase activity was detected by *in-gel* kinase assays (top panel). OST1/SnRK2.6-GFP proteins were detected by immuno-blot using GFP antibody (bottom panel). **c**, OST1-GFP (S171E) kinase activity was tested as shown in (**b**).

a

Peptide	Control	ABA
SSVLHS(+79.97)QPKS(+79.97)TVGTPAYIAPEVLLK	0	1.09E+05

b

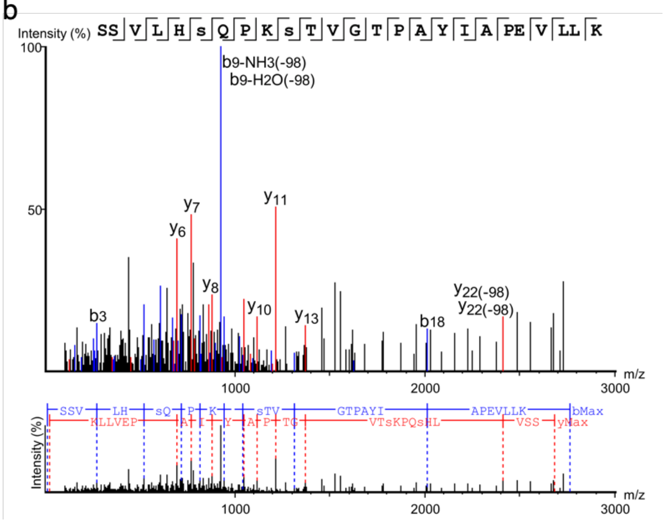

Supplementary Figure 6 | ABA induces phosphorylation at Ser-171 in OST1/SnRK2.6.
a, The sequence of identified phosphorylated peptide by mass spectrometry. OST1/SnRK2.6-GFP was expressed in *Arabidopsis* mesophyll cell protoplasts and purified by immunoprecipitation with GFP antibodies before or after 20 µM ABA treatment for 15 min. S(+79.97) indicates phosphorylated serine residues. Values (Control and ABA) indicate normalized peak areas for phosphorylated peptides. The phosphorylated peptide was not detected in the control sample. In contrast the peptide was clearly phosphorylated in response to ABA. **b**, An annotated mass spectrum of the phosphorylated peptide in the presence of ABA is shown.

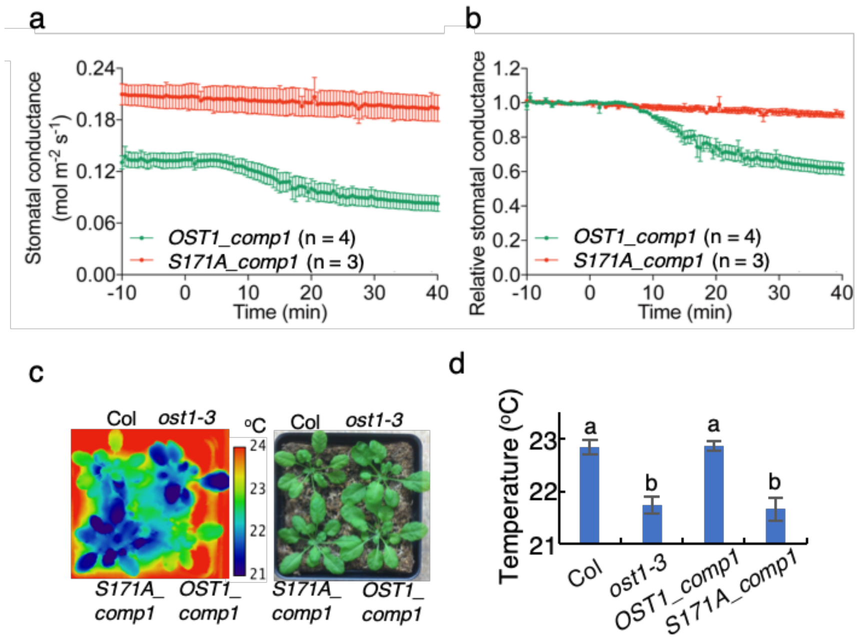

Supplementary Figure 7 | Stomatal conductances and leaf temperatures of independent transgenic *ost1-3 Arabidopsis* lines expressing OST1(WT or S171A)-HF.

a, Stomatal conductances were analyzed in detached leaves of stable transgenic *Arabidopsis* [*pUBQ10:OST1-HF/ost1-3* (*OST1_comp1*) *or* *pUBQ10:OST1(S171A)-HF/ost1-3* (*S171A_comp1*)]. 2 µM ABA was applied at 0 min. Different independent transgenic lines from those shown in Fig. 3a were used. **b**, Relative stomatal conductances in (**a**) normalized to the average of the 10 minutes before addition of ABA. Data presented are mean ± s.e.m. from n = 3 to 4 leaves from independent plants in each genotype. **c**, Leaf temperatures of homozygous transgenic *Arabidopsis* lines (*OST1_comp1* and *S171A_comp1*) were measured by thermal imaging. Plants were sprayed with 20 µM ABA, and thermal images were taken after 3 hr. The bright field image shows where leaves from neighboring plants over-lapped. **d**, Leaf temperatures were measured by using Fiji software (n = 5 experiments, means +/- s.e.m.). Letters at the top of columns are grouped based on one-way ANOVA and Tukey's test, *P*<0.05.

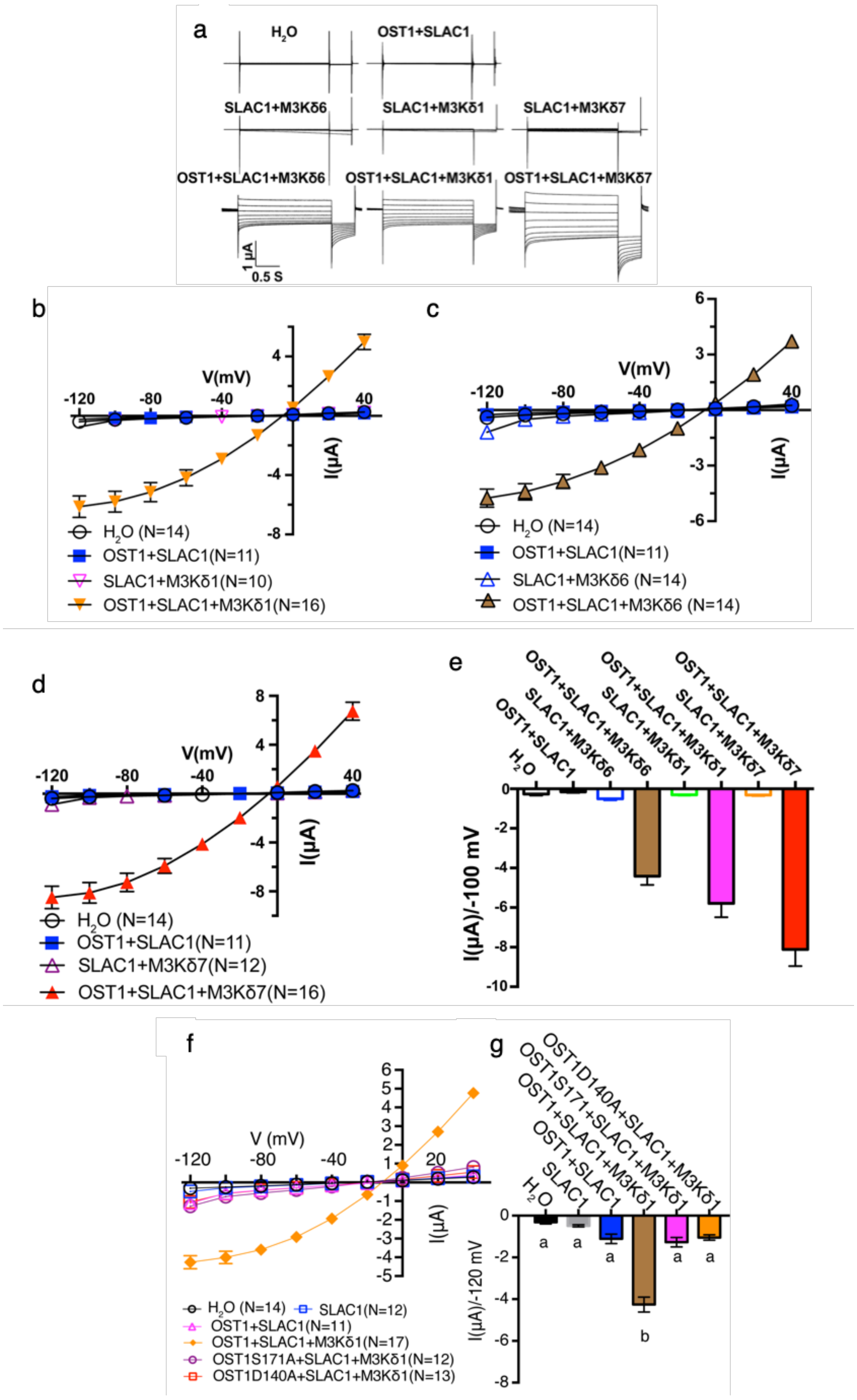

a
H2O
OST1+SLAC1
SLAC1+M3Kδ6
SLAC1+M3Kδ1
SLAC1+M3Kδ7
OST1+SLAC1+M3Kδ6
OST1+SLAC1+M3Kδ1
OST1+SLAC1+M3Kδ7
1 μA
0.5 S
b
V(mV)
I(μA)
H2O (N=14)
OST1+SLAC1(N=11)
SLAC1+M3Kδ1(N=10)
OST1+SLAC1+M3Kδ1(N=16)
c
V(mV)
I(μA)
H2O (N=14)
OST1+SLAC1(N=11)
SLAC1+M3Kδ6 (N=14)
OST1+SLAC1+M3Kδ6 (N=14)
d
V(mV)
I(μA)
H2O (N=14)
OST1+SLAC1(N=11)
SLAC1+M3Kδ7(N=12)
OST1+SLAC1+M3Kδ7(N=16)
e
H2O
OST1+SLAC1
SLAC1+M3Kδ6
OST1+SLAC1+M3Kδ6
SLAC1+M3Kδ1
OST1+SLAC1+M3Kδ1
SLAC1+M3Kδ7
OST1+SLAC1+M3Kδ7
I(μA)/-100 mV
f
V (mV)
I(μA)
H2O (N=14)
SLAC1(N=12)
OST1+SLAC1(N=11)
OST1+SLAC1+M3Kδ1(N=17)
OST1S171A+SLAC1+M3Kδ1(N=12)
OST1D140A+SLAC1+M3Kδ1(N=13)
g
H2O
SLAC1
OST1+SLAC1
OST1+SLAC1+M3Kδ1
OST1S171+SLAC1+M3Kδ1
OST1D140A+SLAC1+M3Kδ1
I(μA)/-120 mV
a
b

Supplementary Figure 8 | M3Kδs activate SLAC1 channels together with OST1/SnRK2.6 in *Xenopus* oocytes

a, Representative whole cell chloride current recordings of oocytes injected with the indicated cRNAs. Currents were recorded in response to voltage pulses ranging from +40 mV to – 120 mV in -20 mV steps with a holding potential at 0 mV and a final tail potential of -120 mV. **b**-**d**, Mean current-voltage curves of oocytes co-expressing OST1 and SLAC1, in the presence or absence of the indicated M3K proteins. In panel (**b**) and (**d**), the symbols of H_2O, OST1+SLAC1 and SLAC1+M3Ks overlapped. One symbol is shown for some data points for better viewing. (**e**) Average SLAC1 mediated currents at -100 mV, co-expressing OST1, in the presence or absence of the indicated M3K proteins. M3Kδ and OST1 and/or SLAC1 cRNA were injected at a concentration ratio of 1 to 10 to 10 (see main text). Data from one representative batch of oocytes are shown, with the number of oocytes in that batch indicated in parentheses. Control H_2O and OST1+SLAC1 data are the same data in (**b**), (**c**) and (**d**), as these data are from the same batch of oocytes (see Methods). Four independent batches of oocytes showed similar results. Error bars denote mean ± s.e.m. **f**, Mean current-voltage curves of *Xenopus* oocytes injected with the indicated cRNAs. **g**, Average SLAC1-mediated currents from panel (**f**) at -120 mV, co-expressing OST1 isoforms, in the presence of M3Kδ1. The injected cRNA ratio of M3Kδ1 and OST1 isoforms was 1 to 10 (see main text). Letters on the bottom of columns are grouped based on one-way ANOVA with Tukey's test, $P < 0.01$.

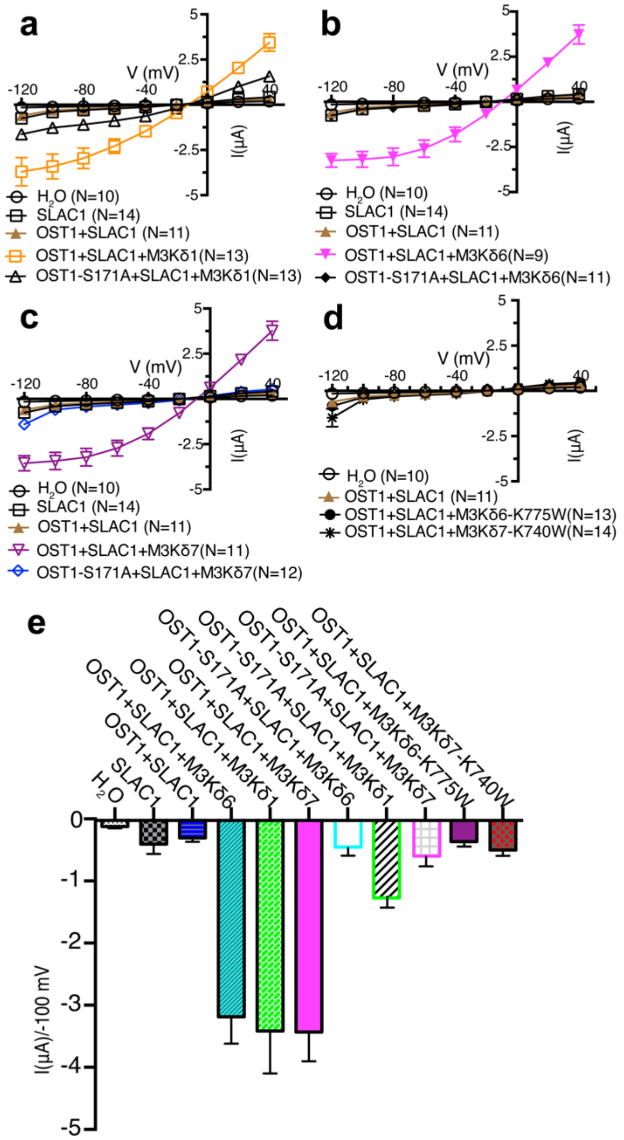

Supplementary Figure 9 | M3K-dependent S-type anion channel activation is dependent on the M3K kinase activities and the Ser171 residue in the OST1/SnRK2.6 activation loop. **a**-**d**, Mean current-voltage curves of *Xenopus* oocytes injected with the indicated cRNAs. The symbols of OST1-S171A+SLAC1+ M3Kδ6 are not visible because the symbols overlap. **e**, Average SLAC1-mediated currents from panels (**a**) to (**d**) at -100 mV, co-expressing OST1 or the kinase inactive OST1-S171A mutant isoform, in the presence of the indicated M3Ks or the kinase-inactive M3Kδ6-K775W and M3Kδ7-K740W isoforms. When M3Kδ and OST1 cRNAs were co-injected the ratio of M3Kδ and OST1 cRNA was 1 to 10 (see main text). Data from one representative oocyte batch are shown. Results from 3 independent batches of oocytes showed similar results. H_2O, SLAC1 and OST1+SLAC1 controls are the same in panels (**a**), (**b**), (**c**) and (**d**), as these were recorded in the same batch of oocytes. Error bars denote mean ± s.e.m.

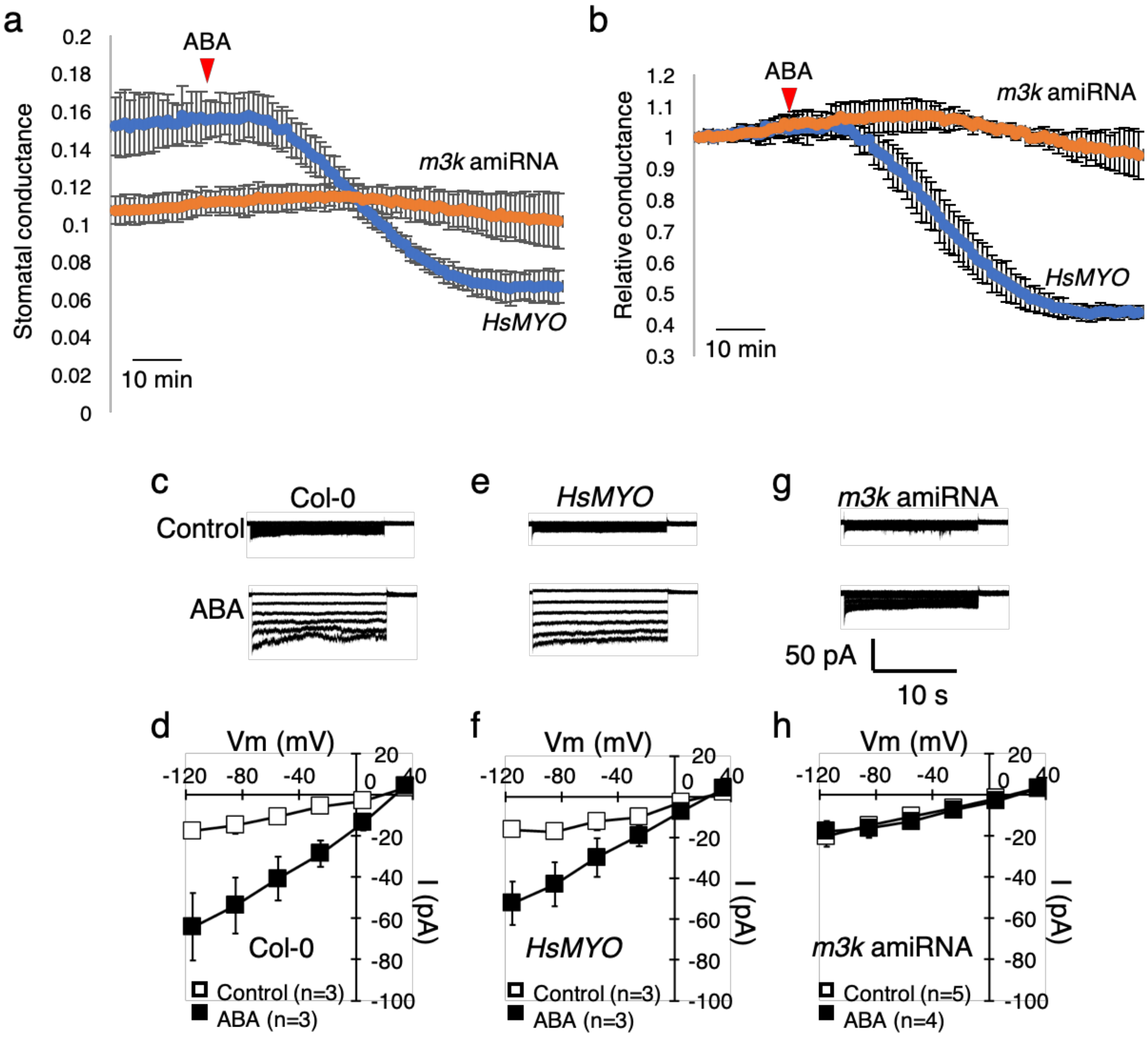

Supplementary Figure 10 | ABA-induced stomatal closing and activation of guard cell S-type anion channels are impaired *m3k* amiRNA line.

a, Leaves from *m3k* amiRNAi and the control *amiRNA-HsMYO* line (expressing an amiRNA targeting human *myosin 2*), which has no target gene in *Arabidopsis* plants were analyzed in time-resolved stomatal conductance analyses in which 1 µM ABA was added to the transpiration stream via the petiole[3] as indicted by the red arrowhead (n = 3 leaves from 3 independent plants per genotype, +/- s.d.). **b**, Normalized relative stomatal conductance to the first data point shown in (**a**). **c-h**, ABA-activated S-type anion channel currents were investigated by patch-clamp analyses using guard cell protoplasts from the wildtype parent Col-0 (**c**, **d**), the *HsMYO* amiRNA control line (**e**, **f**), and the *m3k* amiRNA line (**g**, **h**). Representative current traces (**c**, **e**, **g**) and average current voltage relationships (**d**, **f**, **h**) of S-type anion channel currents are shown. Data presented are means +/- s.e.m.

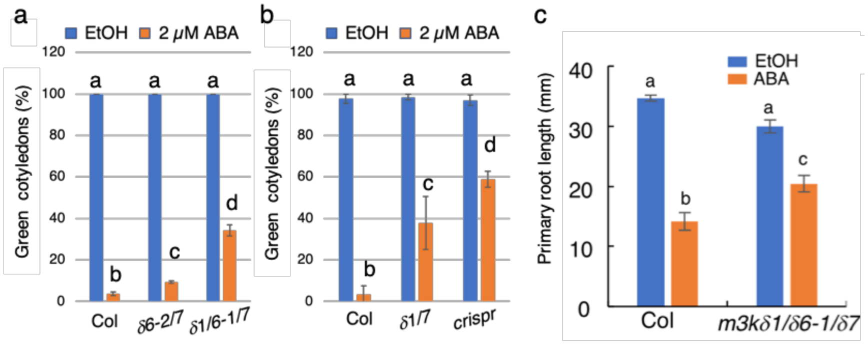

Supplementary Figure 11 | *m3k* double mutants show weak ABA-insensitive phenotypes.

a, *m3k* double *(m3kδ6-2/δ7)* and triple *(m3kδ1/δ6-1/δ7)* mutants were grown on 1/2 MS plates supplemented with 2 µM ABA or EtOH for 9 days. Seedlings showing green cotyledons were counted. n = 3 experiments, means +/- s.d., 45-48 seeds were used per genotype and condition in each experiment. **b**, *m3k* double *(m3kδ1/δ7)* and triple *(m3kδ1crispr/δ6-2/δ7crispr)* mutants were grown on 1/2 MS plates supplemented with 2 µM ABA or EtOH for 16 days. Seedlings showing green cotyledons were counted. n = 3 experiments (EtOH) and n = 4 experiments (ABA), means +/- s.d., 45 seeds were used per genotype and condition in each experiment. The *crispr* and Col results are the same as Figure 5e because they were grown on the same plates. Letters at the top of columns are grouped based on two-way ANOVA and Tukey's test, $P<0.05$. **c**, Wild type and *m3kδ1/δ6-1/δ7* triple mutant seedlings were grown on 1/2 MS plates for three days and transferred to 1/2 MS plates with or without 20 µM ABA followed by an additional seven-day incubation. Primary root length was measured using ImageJ software. n = 5 experiments, means +/- s.e.m., 10-13 seedlings per genotype were used in each experiment. Letters at the top of columns are grouped based on two-way ANOVA and Tukey's test, $P<0.05$.

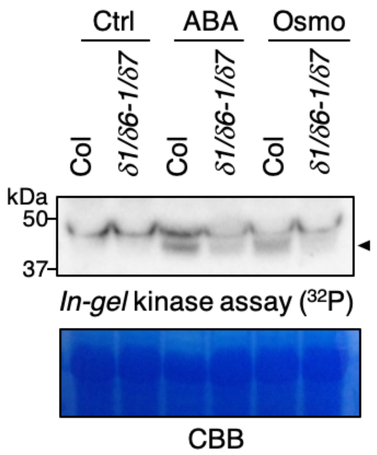

Supplementary Figure 12 | Biological replicate example of SnRK2 activities in *m3kδ1/δ6-1/δ7* triple mutant line.

Replicate of *in-gel* kinase assay using *m3kδ1/δ6-1/δ7* triple mutant is shown. See Figure 6c for an independent experiment. Arrowhead indicates SnRK2 kinase activities.

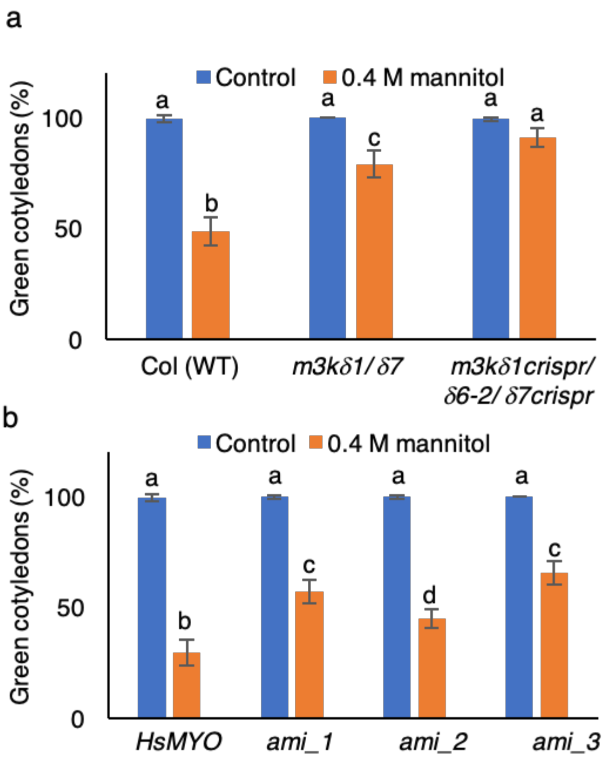

Supplementary Figure 13 | *m3k* mutants show reduced sensitivity to osmotic stress in seed germination.

a, *m3kδ1/δ7* double mutant and *m3kδ1crispr/δ6-2/δ7crispr* triple mutant seedlings were grown on 1/2 MS plates supplemented with 0.4 M mannitol for 3 days. Green cotyledons were counted. n = 4 experiments, means +/- s.d., 64 seeds per genotype were used in each experiment. **b**, Three amiRNA lines targeting *M3Kδ1, δ6* and δ*7* were grown on 1/2 MS plate supplemented with 0.4 M mannitol for 3 days. As a control line, *HsMYO* was used. n = 4 experiments, means +/- s.d., 64 seeds per genotype were used in each experiment. Letters at the top of columns are grouped based on two-way ANOVA and Tukey's test, $P<0.05$.

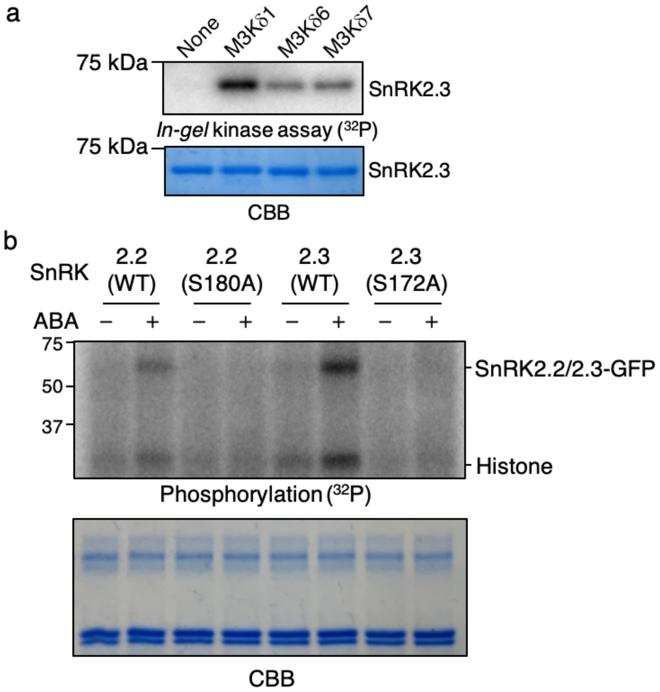

Supplementary Figure 14 | M3Ks activate SnRK2.3 *in vitro*.
a, GST-SnRK2.3 protein was incubated with the kinase domains of M3Kδ1, M3Kδ6 or M3Kδ7 and *in-gel* kinase assays were conducted. **b**, SnRK2.2-GFP (WT or S180A) and SnRK2.3-GFP (WT or S172A) were expressed in *Arabidopsis* mesophyll cell protoplasts and purified by immunoprecipitation with GFP antibodies. The isolated proteins bound on magnetic immunoprecipitation beads were used for *in vitro* phosphorylation assays using histone as an artificial substrate. Phosphorylation reactions were started by the addition of ^{32}P-ATP. After 30 min, reactions were stopped by the addition of 3xSDS-PAGE sample buffer.

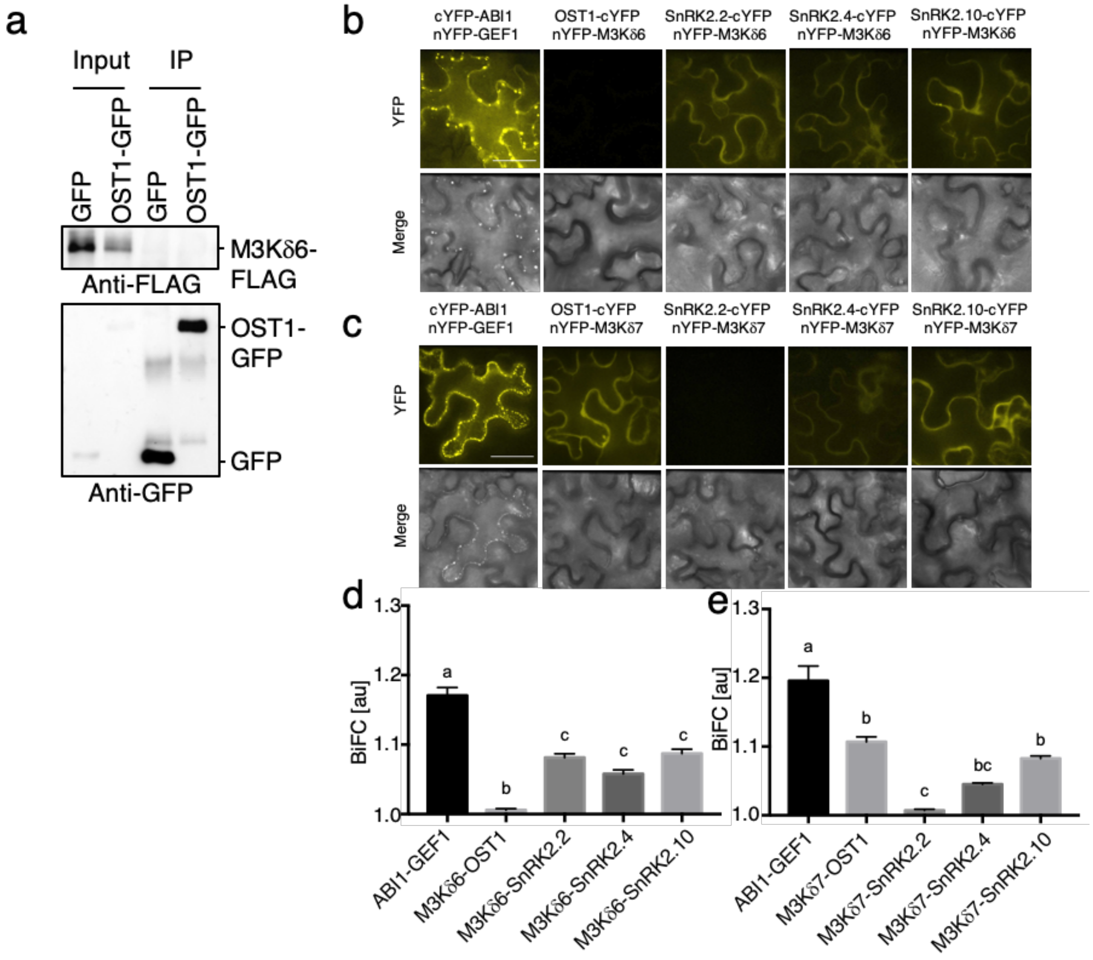

Supplementary Figure 15 | M3Ks interact with SnRK2 kinases in BiFC experiments in plant cells.

a, Co-immunoprecipitation experiments using transiently expressed M3Kδ6 and OST1/SnRK2.6 in *Arabidopsis* mesophyll cell protoplasts. OST1/SnRK2.6-GFP or GFP control co-expressed with M3Kδ6-FLAG were immunoprecipitated with GFP antibodies. Precipitated proteins were analyzed by immunoblots using GFP or FLAG antibody. **b** and **c**, BiFC analyses of nYFP-M3Kδ6 (**b**) or nYFP-M3Kδ7 (**c**) with OST1/SnRK2.6-cYFP, SnRK2.2-cYFP, 2.4-cYFP and 2.10-cYFP infiltrated in 6-week-old *Nicotiana benthamiana* leaves. nYFP-GEF1 / cYFP-ABI1 combination was used as a positive control. All images are at the same scale. Scale bars = 50 µm. **d** and **e**, BiFC quantifications measured from maximal projections of z-stacks and normalized over an infiltration control expressing p19 only. BiFC quantifications were analyzed by one-way ANOVA followed by Tukey's tests. Confocal images were acquired using identical settings for each BiFC experiment. Means ± s.e.m. (n = 45).

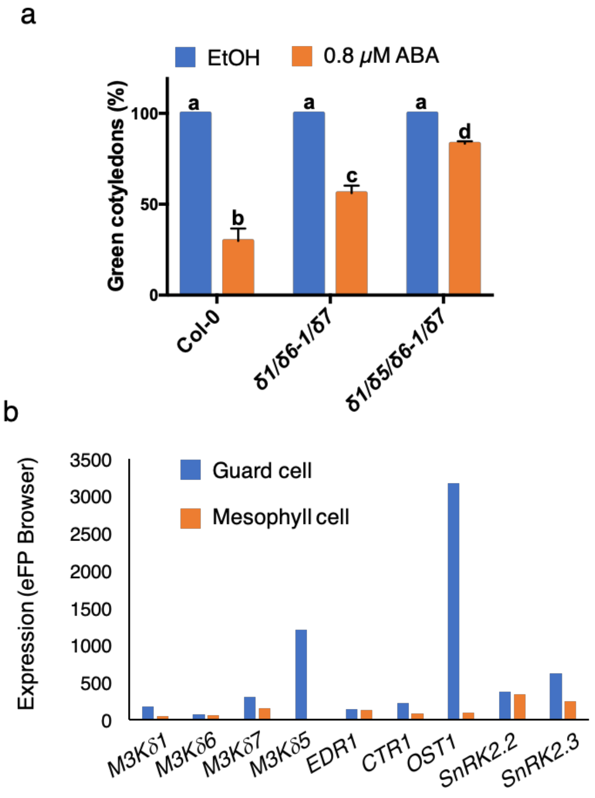

Supplementary Figure 16 | *m3k* quadruple mutant shows an ABA-insensitive phenotype in cotyledon emergence.

a, *m3k* triple *(m3kδ1/δ6-1/δ7)* and *m3k* quadruple (*m3kδ1/δ5/δ6-1/δ7*) mutant plants were grown on 1/2 MS plates supplemented with 0.8 µM ABA for 6 days (*m3kδ5* = SALK_025685). Green emerging cotyledons were counted. n = 6 experiments, means +/- s.d., 81 seeds were used per genotype and per condition in each experiment. Letters at the top of columns are grouped based on two-way ANOVA and Tukey's test, $P<0.05$. **b**, Gene expression levels of B3 subgroup *M3K* genes and three *SnRK2* genes in guard cells and mesophyll cells. Data were obtained from the public microarray database eFP Browser (http://bar.utoronto.ca/efp/cgi-bin/efpWeb.cgi)[4].

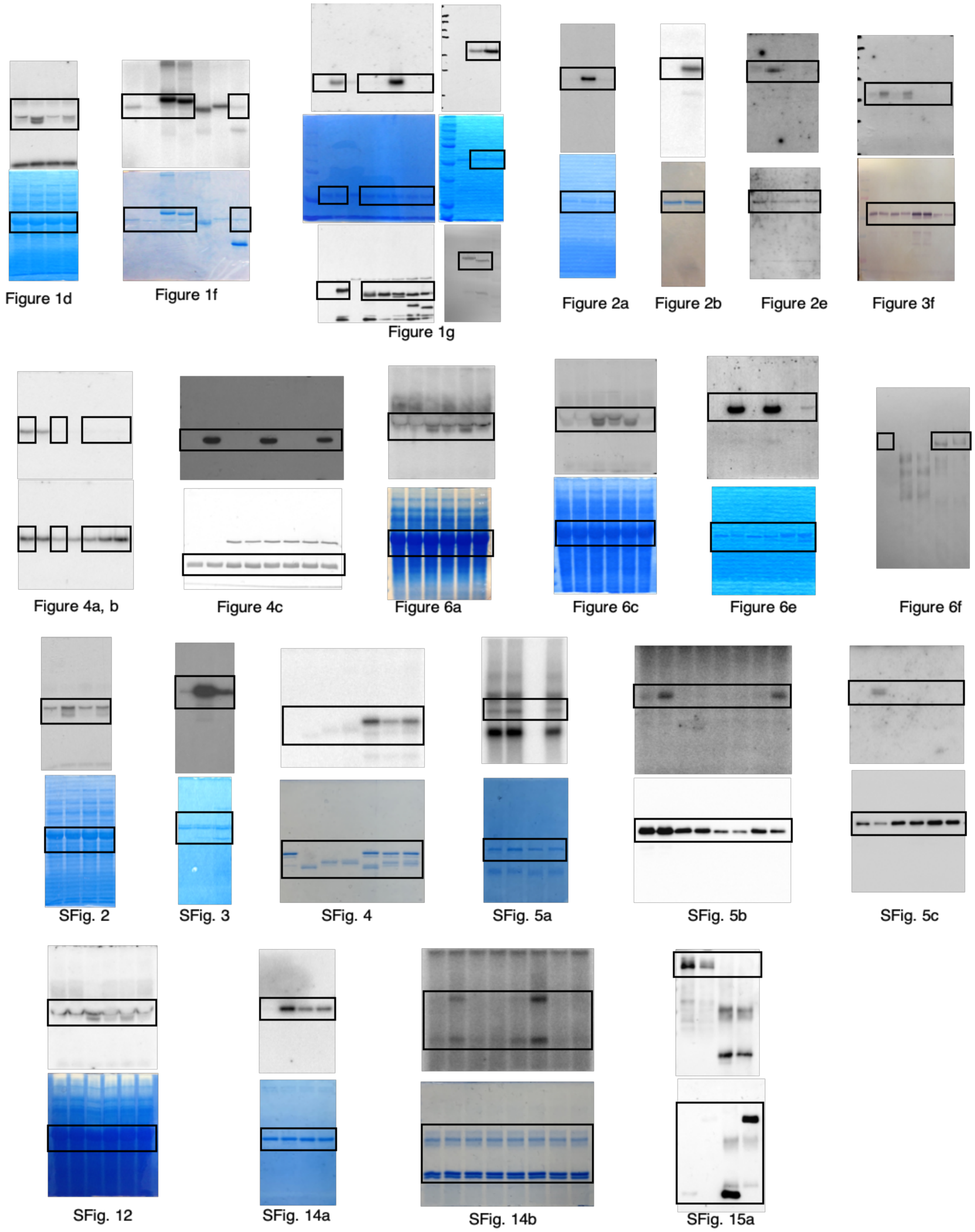

Supplementary Figure 17 | Uncropped gel and blot images presented in this study. Cropped regions are shown by boxes.

For vector constructions

M3Kdelta1KD_USER_F56	ggcttaauatgGAAGCTGCAAAGTGGGAAATTATG
M3Kdelta1_USER_R55	ggtttaauccTTACATCAAACTTTCACTCGTATTC
M3Kdelta7KD_USER_F52	ggcttaauGCTGCAATTGATGATGTAG
M3Kdelta7_USER_R52	ggtttaauccTTATTGTTCCTGCTCATCAG
M3Kdelta6KD_USER_F52	ggcttaauGATGTATCTGACTGTGAG
M3Kdelta6_USER_R52	ggtttaauccTCAATCCTTTTGTTCATGTTCAG
M3Kdelta5KD_USER_F52	ggcttaauAAAGGAAGGTTACCTGTTG
M3Kdelta5_USER_R52	ggtttaauccCTATAGTATGGGAGCTGATTTAG
EDR1KD_USER_F55	ggcttaauatgGATGTTGGTGAATGTGAAATTCC
EDR1_USER_R56	ggtttaauccCTATTGTGGTGTAGGAAGTACAAG
1g16270KD_USER_F56	ggcttaauatgTCAGAAACAAGGAACGCTGG
1g16270_USER_R56	ggtttaauccTCACTTTGAAGCCTTGTGAGC
2g42640KD_USER_F54	ggcttaauatgAGCCACATTTGGAATGAAGTG
2g42640_USER_R56	ggtttaauccTCAGCAAATCGGAATCTCGC
M3Kdelta1_USER_F54	ggcttaauATGTCGAAGATGAAGCATCTTC
M3Kdelta1-stop.user.R53	ggtttaauCATCAAACTTTCACTCGTATTC
M3Kdelta6_USER_F52	ggcttaauATGAAAGTAAAAGAAGAAACTTTG
M3Kdelta6-stop.user.R53	ggtttaauATCCTTTTGTTCATGTTCAGTTG
M3Kdelta7_USER_F53	ggcttaauATGAAGATGAACATGAAGAAATTTC
M3Kdelta7-stop.user.R54	ggtttaauTTGTTCCTGCTCATCAGTG
M3Kdelta1_InF_pET.F54	gctgatatcggatccATGTCGAAGATGAAGCATCTTC
M3Kdelta1_InF_pET.R54	cggagctcgaattcTTACATCAAACTTTCACTCGTATTC
OST1_USER_F56	ggcttaauATGGATCGACCAGCAGTG
OST1_USER_R56	ggtttaauCATTGCGTACACAATCTCTCC
SnRK2.2_USER_F56	ggcttaauATGGATCCGGCGACTAATTC
SnRK2.2_USER_R55	ggtttaauGAGAGCATAAACTATCTCTCCAC
SnRK2.3_USER_F58	ggcttaauATGGATCGAGCTCCGGTG
SnRK2.3_USER_R56	ggtttaauGAGAGCGTAAACTATCTCTCC
SnRK2.4_USER_F55	ggcttaauATGGACAAGTACGAGCTGG
SnRK2.4_USER_R55	ggtttaauACTTATTCTCACTTCTCCACTTG
SnRK2.10.user.F57	ggcttaauATGGACAAGTACGAGCTTGTTAAAG
SnRK2.10.user.R54	ggtttaauTTAACTGACTCGGACTTCTC

For RT-PCR

M3Kdelta1.F54	ATGTCGAAGATGAAGCATCTTC
M3Kdelta1.R54	TTACATCAAACTTTCACTCGTATTC
M3Kdelta6.F52	ATGAAAGTAAAAGAAGAAACTTTG
M3Kdelta6_KD_F52	ATGGTTGCTGCTGCTG
M3Kdelta6.R52	TCAATCCTTTTGTTCATGTTCAG
M3Kdelta7.F53	ATGAAGATGAACATGAAGAAATTTC
M3Kdelta7.R52	TTATTGTTCCTGCTCATCAG
ACT8.Fw58	CACCCGGTTCTACTTACCGA
ACT8.Rv58	AGACGGAGGATAGCATGTGG

Supplementary Table 1 | Primer sequences used in this study.

Reference

1 MAPK Group. Mitogen-activated protein kinase cascades in plants: a new nomenclature. *Trends Plant Sci* **7**, 301-308 (2002).

2 Belin, C. *et al.* Identification of features regulating OST1 kinase activity and OST1 function in guard cells. *Plant Physiol* **141**, 1316-1327, doi:10.1104/pp.106.079327 (2006).

3 Ceciliato, P. *et al.* Intact leaf gas exchange provides a robust method for measuring the kinetics of stomatal conductance responses to abscisic acid and other small molecules in Arabidopsis and grasses. *Plant Methods* **15**, doi:10.1186/s13007-019-0423-y (2019).

4 Yang, Y., Costa, A., Leonhardt, N., Siegel, R. S. & Schroeder, J. I. Isolation of a strong Arabidopsis guard cell promoter and its potential as a research tool. *Plant Methods* **4**, 6, doi:10.1186/1746-4811-4-6 (2008).
