# Supplementary material for: MAP3Kinase-dependent SnRK2-kinase activation is required for abscisic acid signal transduction and rapid osmotic stress response
Source: Nat Commun. 2020 Jan 2;11:12. doi: 10.1038/s41467-019-13875-y (PMC6940395; doi:10.1038/s41467-019-13875-y)
Supplement: Supplementary file 3 — Reporting Summary [file 41467_2019_13875_MOESM3_ESM.pdf]

nature research

Corresponding author(s): Julian I. Schroeder

Last updated by author(s): Nov 27, 2019

Reporting Summary

Nature Research wishes to improve the reproducibility of the work that we publish. This form provides structure for consistency and transparency in reporting. For further information on Nature Research policies, see Authors & Referees and the Editorial Policy Checklist .

Statistics

For all statistical analyses, confirm that the following items are present in the figure legend, table legend, main text, or Methods section.

n/a	Confirmed	
☐	☒	The exact sample size (*n*) for each experimental group/condition, given as a discrete number and unit of measurement
☐	☒	A statement on whether measurements were taken from distinct samples or whether the same sample was measured repeatedly
☐	☒	The statistical test(s) used AND whether they are one- or two-sided<br>*Only common tests should be described solely by name; describe more complex techniques in the Methods section.*
☒	☐	A description of all covariates tested
☒	☐	A description of any assumptions or corrections, such as tests of normality and adjustment for multiple comparisons
☐	☒	A full description of the statistical parameters including central tendency (e.g. means) or other basic estimates (e.g. regression coefficient) AND variation (e.g. standard deviation) or associated estimates of uncertainty (e.g. confidence intervals)
☐	☒	For null hypothesis testing, the test statistic (e.g. *F*, *t*, *r*) with confidence intervals, effect sizes, degrees of freedom and *P* value noted<br>*Give P values as exact values whenever suitable.*
☒	☐	For Bayesian analysis, information on the choice of priors and Markov chain Monte Carlo settings
☒	☐	For hierarchical and complex designs, identification of the appropriate level for tests and full reporting of outcomes
☒	☐	Estimates of effect sizes (e.g. Cohen's *d*, Pearson's *r*), indicating how they were calculated

Our web collection on statistics for biologists contains articles on many of the points above.

Software and code

Policy information about availability of computer code

Data collection	Software was used depending on the specific methods used for each figure panel (pclamp, image, Fiji, Licor gas exchange analyzer, Excel, FLIR, Mass Spectrometry data sets), Metamorph version 7.7.7.0.
Data analysis	Fiji software (ImageJ version: 2.0.0-rc-59/1.51n), pclamp, Excel, FLIR, MS/MS spectra analyses, WMD3 (http://wmd3.weigelworld.org/cgi-552 bin/webapp.cgi), PHANTOM database (http://phantomdb.ucsd.edu), Metamorph version 7.7.7.0

For manuscripts utilizing custom algorithms or software that are central to the research but not yet described in published literature, software must be made available to editors/reviewers. We strongly encourage code deposition in a community repository (e.g. GitHub). See the Nature Research guidelines for submitting code & software for further information.

Data

Policy information about availability of data

All manuscripts must include a data availability statement. This statement should provide the following information, where applicable:
- Accession codes, unique identifiers, or web links for publicly available datasets
- A list of figures that have associated raw data
- A description of any restrictions on data availability

All relevant data and Arabidopsis transgenic lines used in this study are available from the corresponding author upon request.

Field-specific reporting

Please select the one below that is the best fit for your research. If you are not sure, read the appropriate sections before making your selection.

Life sciences study design

All studies must disclose on these points even when the disclosure is negative.

Sample size	Sample sizes were chosen based on previous related studies in the biological research area and effect size.
Data exclusions	No data were excluded.
Replication	The number of independent repeat experiments is given in the manuscript and figure legends for experiments.
Randomization	No specific experimental sequence was selected when comparing genotypes, enabling a degree of randomization.
Blinding	Experiments were not blinded in this study.

Reporting for specific materials, systems and methods

We require information from authors about some types of materials, experimental systems and methods used in many studies. Here, indicate whether each material, system or method listed is relevant to your study. If you are not sure if a list item applies to your research, read the appropriate section before selecting a response.

Materials & experimental systems

n/a	Involved in the study	
☐	☒	Antibodies
☒	☐	Eukaryotic cell lines
☒	☐	Palaeontology
☒	☐	Animals and other organisms
☒	☐	Human research participants
☒	☐	Clinical data

Methods

n/a	Involved in the study	
☒	☐	ChIP-seq
☒	☐	Flow cytometry
☒	☐	MRI-based neuroimaging

Antibodies

Antibodies used	Anti-FLAG M2 antibody produced in mouse (Sigma)<br>Anti-GFP Ab-1 (Clone GFP01) (Thermo Scientific)<br>Anti-His (MA1-4806) (Thermo Scientific)<br>Anti-GST (27457701) (GE Healthcare)
Validation	https://www.sigmaaldrich.com/catalog/product/sigma/f1804?lang=en&region=US<br>https://assets.fishersci.com/TFS-Assets/APD/Specification-Sheets/D12167~.pdf<br>https://www.thermofisher.com/antibody/product/6x-His-Tag-Antibody-clone-13-45-31-2-Monoclonal/MA1-4806<br>https://www.gelifesciences.com/en/us/shop/chromatography/resins/affinity-tagged-protein/anti-gst-antibody-p-06000
